# Supplementary material for: Elucidating the immune infiltration in acne and its comparison with rosacea by integrated bioinformatics analysis
Source: PLoS One. 2021 Mar 24;16(3):e0248650. doi: 10.1371/journal.pone.0248650 (PMC7990205; doi:10.1371/journal.pone.0248650)
Supplement: S1 File — (DOCX) [file pone.0248650.s001.docx]

**Availability of data and materials**

The datasets generated and analyzed during the current study are available in the GEO (https://www.ncbi.nlm.nih.gov/geo/) repository, GSE108110 (https://www.ncbi.nlm.nih.gov/geo/query/acc.cgi?acc=GSE108110), GSE53795 (https://www.ncbi.nlm.nih.gov/geo/query/acc.cgi?acc=GSE53795), GSE65914 (https://www.ncbi.nlm.nih.gov/geo/query/acc.cgi?acc=GSE65914), GSE14905 (https://www.ncbi.nlm.nih.gov/geo/query/acc.cgi?acc=GSE14905), and GSE78097 (https://www.ncbi.nlm.nih.gov/geo/query/acc.cgi?acc=GSE78097) datasets.
