# Supplementary material for: Elucidating the immune infiltration in acne and its comparison with rosacea by integrated bioinformatics analysis
Source: PLoS One. 2021 Mar 24;16(3):e0248650. doi: 10.1371/journal.pone.0248650 (PMC7990205; doi:10.1371/journal.pone.0248650)
Supplement: Attachment — Submitted filename: 20201221 Immune Infiltration in Acne review.docx [file pone.0248650.s002.docx]

Review for PLOS One Manuscript

“Elucidating the immune infiltration in acne and its comparison with rosacea by integrated bioinformatics analysis”

Submitted 12/22/20

The manuscript by Yang and colleagues investigates differential immune infiltration in common inflammatory conditions of facial skin. Using bioinformatics approaches, the authors seek out how these different pathologies may recruit, promote, or repress different immune cell subsets. Important questions the study attempts to answer are (1) How does immune cell infiltration differ between common conditions of acne vulgaris and rosacea, (2) how does immune cell infiltration differ in the lesions of acne patients compared to the unaffected skin in those same patients, and (3) are there immune components highly specific to acne lesions that may serve as immunotherapeutic targets. The authors draw upon publicly available microarray datasets of whole skin biopsies from patients with acne vulgaris, different forms of rosacia, and healthy controls. The authors first remove batch effects and normalize between datasets then apply the commonly used CIBERSORT algorithm in order to deconvolute the bulk microarray data in order to gain insight into different immune cell infiltrates. Key findings unique to active acne lesions include higher neutrophil, monocyte, and activated mast cell infiltration relative to other conditions. Given the previous characterization of Th1/Th17 imbalance in inflammatory skin conditions this study finds that the relative recruitment of T cells is largely unaltered. Interestingly, the authors also find that non-lesional skin of acne patients typically have a higher rate of Treg infiltration. Because the CIBERSORT algorithm does not parse out different T cell subsets, the authors conclude by assessing relative expression of T helper cell subsets, Th1 and Th17 related molecules, and find that they are elevated, agreeing with prior findings. The study is fundamentally strong and seeks to answer important questions in the field. With some additional analysis and clarification of comments below, I would recommend the study for publication.

Major Comments

1. The authors use the CIBERSORT algorithm to great effect and it reveals much about the differences in immune cell recruitment between different inflammatory pathologies about the skin. They fail, however, to go further with the dataset they have at their disposal. For example, the study identifies activated mast cells as one of the populations recruited higher in lesional acne relative to other conditions and then goes on in the discussion (lines 258-262) and mentions molecules implicated in the recruitment of mast cells. The authors could simply screen expression of these molecules in the analyzed datasets to determine whether they are partially responsible for immune cell recruitment.
2. Another fundamental problem with the study is that it largely focuses on immune cell infiltration of these different pathologies, but then only reviews the relative proportions of immune cells to each other. The study needs to also cover what proportion of the samples were immune cells relative to other cell types (eg epithelial cells, endothelial cells etc.). This can be accomplished by either contacting originators of the original datasets for the most accurate data, or if not possible they can contact other clinicians working in the field or others with relevant animal models.

Minor Comments

1. Lines 83-84 are these lesions and non-lesions from the same patients?
2. Figure 2A and 2B include 2-dimensional PCA plots for ease of visualization
3. Figure 2A and 2B include %variance explained by each principal component
4. Lines 108-109 p-value attained from differential expression or from CIBERSORT algorithm? Please specify
5. Line 122 typo is “valuated” should be “evaluated”
6. Does CIBERSORT tell you what proportion of TOTAL cells biopsied are immune or hematopoietic? Here we see relative proportions of immune subsets which may be misleading if there are vastly different proportions of immune infiltration to begin with.
7. Figures 3A and 3B need labelling to identify which datasets correspond to which groups of patients, similar to figures 3a and 3b
8. 3C and 3D should flip cluster heatmaps so that “healthy patients” are in the same side of the figure, this can be done easily in R by ordering the samples how they naturally cluster, then turning off the cluster columns in the pheatmap function
9. Some more intro as to why it is important to compare acne and rosacea… are they commonly associated/treated?
10. Figure 4 needs to depict what the numeric values are in reference to. To me it seems as though they are R^2 values but it should be clearer. Additionally, p-values for the correlations referred to in lines 166-168. Lastly, is this correlation matrix constructed from within the acne <48hrs group? The text speaks about a comparison between acne <48hrs and healthy controls which makes it confusing as to what data is used to construct the correlation matrix.
11. What data is the PCA cluster analysis based off? If it is based off total gene expression data and not just the CIBERSORT data, then it is misleading to stat that it is comparison in immune cell infiltration.
12. Lines 199-200 comment on infiltration of Th1/Th17 cells but the authors are looking at expression of molecules that are expressed by multiple cell-types therefore it is inaccurate to make the claim that they are assessing Th1/Th17 infiltration
13. From 271-285 it is clear that skin immunology and facial skin immunology at the steady state are not discussed. This can be covered either in the introduction or the discussion but the authors should discuss what sorts of immune cells are normally present in the skin or in the facial skin in particular and in what proportions. This is covered partially through their computational work but they should also cover the prior literature.
14. Another possibility to explain the dearth of activated immune cells in non-lesional skin of acne patients is the possibility that immune cells that are normally evenly distributed throughout the skin (eg in healthy patients examined in the study) traffic into lesions and leave the non-lesional skin immune depleted. The authors can cover this in their discussion if appropriate.
